# Insights into cognitive and behavioral comorbidities of *SLC6A1*-related epilepsy: five new cases and literature review

**DOI:** 10.3389/fnins.2023.1215684

**Published:** 2023-08-28

**Authors:** Marina Trivisano, Ambra Butera, Chiara Quintavalle, Angela De Dominicis, Costanza Calabrese, Simona Cappelletti, Federico Vigevano, Antonio Novelli, Nicola Specchio

**Affiliations:** ^1^Clinical and Experimental Neurology, Bambino Gesù Children’s Hospital, IRCCS, Full Member of European Reference Network EpiCARE, Rome, Italy; ^2^Unit of Child Neurology and Psychiatry, Department of Human Pathology of the Adult and Developmental age “Gaetano Barresi”, University of Messina, Messina, Italy; ^3^PROMISE Department, School of Child Neurology and Psychiatry, University of Palermo, Palermo, Italy; ^4^Laboratory of Medical Genetics, Translational Cytogenomics Research Unit, Bambino Gesù Children’s Hospital, IRCCS, Rome, Italy

**Keywords:** *SLC6A1*, genetic epilepsy, developmental and epileptic encephalopathy, neuropsychological features, autism spectrum disorder

## Abstract

**Introduction:**

*SLC6A1* pathogenic variants have been associated with epilepsy and neurodevelopmental disorders. The clinical phenotype includes different seizure types, intellectual disability, and psychiatric symptoms affecting mood and behavior. Few data regarding neuropsychological features have been described, and details on cognitive profiles are often missing due to the lack of standardized tests.

**Methods:**

We retrospectively reviewed the neuropsychological assessments of five subjects carrying heterozygous missense genetic variants in *SLC6A1*. We also collected data on epileptic features, EEGs, and brain MRIs. Additionally, we reviewed neuropsychological data from 204 previously reported patients with *SLC6A1* pathogenic variants.

**Results:**

In our series, at the last evaluation (median 12.6 years), three patients had borderline intellectual functioning, one patient had mild cognitive impairment, and one patient presented with a moderate cognitive disability. Three out of five patients underwent at least two neuropsychological evaluations, which revealed a worsening of cognitive functions over time. We detected attention deficits in all patients. In addition, we observed anxiety, disruptive behavior disorder, emotional instability, and hetero aggressiveness. We also performed a literature review that highlighted that most of the patients with *SLC6A1* pathogenic variants have mild-to-moderate intellectual disability and that one-third of cases have autistic traits.

**Discussion:**

Based on the literature review and the detailed description of our cases, we conclude that patients with *SLC6A1*-related epilepsy mostly present with mild-to-moderate intellectual disability, often associated with attention disorders. Such symptoms may worsen over time. Periodic standardized neuropsychological tests may be useful tools to follow development over time, and patient-specific rehabilitation programs could be tailored consistently.

## Introduction

Solute carrier (SLC) proteins are ubiquitous transmembrane transporters with a crucial role in maintaining the physiological functions of organisms and as medication targets (Pizzagalli et al., 2021; Gan et al., 2022). *SLC6A1* [MIM: 137165] is located on the short arm of chromosome 3 (3p25.3) and encodes sodium chloride-dependent GABA transporter type 1 (GAT-1), a voltage-dependent transmembrane protein (Bröer and Gether, 2012; Kahen et al., 2022). GAT-1 activity is essential for GABA reuptake into the presynaptic terminals of neurons and glia (Bain et al., 2022; Gan et al., 2022).

Pathogenic variants of *SLC6A1* destabilize the global protein conformation, causing a loss of GAT-1 function that leads to alterations in GABA levels in the synaptic cleft (Kahen et al., 2022). This imbalance may independently affect many pathways involved in both neurological and psychiatric diseases, leading to a wide spectrum of clinical phenotypes, including epilepsy, intellectual disability (ID), and behavioral disorders (Bröer and Gether, 2012; Carvill et al., 2015; Poliquin et al., 2021).

*SLC6A1* pathogenic variants were originally detected in patients affected by epilepsy with myoclonic-atonic seizures (EMAtS) (Carvill et al., 2015; Fischer et al., 2022). Later, patients with Lennox–Gastaut Syndrome (LGS) were also reported, expanding the phenotypic spectrum (Cai et al., 2019). Nevertheless, additional authors reported *SLC6A1* pathogenic variants in patients suffering from a heterogeneous spectrum of epilepsies, that did not always meeting criteria for a defined epileptic syndrome (Kalvakuntla et al., 2023). Overall, the most frequent seizure types associated with *SLC6A1* genetic variants are myoclonic-atonic seizures and absences with eyelid myoclonia, followed by tonic–clonic seizures (TCS) (Johannesen et al., 2018; Goodspeed et al., 2020; Tang et al., 2020; Kahen et al., 2022).

In the last few years, a wide range of neurodevelopmental comorbidities have been associated with *SLC6A1*-related epilepsy (Fischer et al., 2022; Kahen et al., 2022). Affected subjects often present with language disorder and cognitive impairment that progresses from developmental delay (DD) or developmental regression to intellectual disability (Johannesen et al., 2018; Kahen et al., 2022; Kalvakuntla et al., 2023). Behavioral disorders such as attention-deficit/hyperactivity disorder (ADHD), aggressivity, anxiety, or mood disorders, as well as other more severe psychiatric features, such as autism spectrum disorder (ASD) and schizophrenia, may contribute to the severity of the clinical phenotype (Johannesen et al., 2018; Goodspeed et al., 2020; Rees et al., 2020; Fischer et al., 2022; Kahen et al., 2022; Kalvakuntla et al., 2023). Additional features such as hypotonia, learning disorders, high pain tolerance, sleep disturbances, and movement disorders have also been reported (Poliquin et al., 2021; Kahen et al., 2022; Verhoeven et al., 2022).

In this study, we describe the neuropsychological profile of five new patients carrying *SLC6A1* pathogenic variants, detailing the cognitive and behavioral features. The clinical presentation of all subjects was consistent with EMAtS. Moreover, we performed a literature review, focusing on the neuropsychological profiles (cognitive and behavioral details), which includes 204 previously published patients with *SLC6A1* pathogenic variants.

## Methods

We retrospectively reviewed the medical charts of five patients with *SLC6A1* pathogenic variants who were followed at Bambino Gesù Children’s Hospital in Rome, Italy. *SLC6A1* pathogenic variants were identified in all patients through a trio-based New Generation Sequencing (NGS) epilepsy gene panel. We collected clinical data, including sex, family history, age at seizure onset, seizure semiology and frequency, intellectual disability, behavioral and psychiatric disorders, EEG and brain MRI.

Seizure types and epilepsy syndromes were classified according to the Commission on Classification and Terminology of the International League Against Epilepsy (Fisher et al., 2017; Scheffer et al., 2017; Specchio et al., 2022).

We retrospectively reviewed all cognitive and behavioral evaluations for all patients performed with the following scales:

1. Developmental and cognitive assessment:
  - *Griffiths Mental Development Scales* (GMDS).
  - *Wechsler Intelligence Scale for Children,* fourth edition (WISC IV).
  - *Wechsler Adult Intelligence Scale*, fourth edition (WAIS-IV).
  - *Wechsler Preschool and Primary Scale of Intelligence,* third edition (WPPSI-III).
  - *Raven’s Progressive Matrix* (RPM): PM38 and PM47.
  - *Leiter- R*
2. Behavioral assessment:
  - *Child Behavior Checklist* (CBCL) which includes eight syndrome scales (anxiety/depression, depression, somatic complaints, social problems, thinking problems, attention problems, rule-breaking behaviors, and aggressive behaviors) that lead to group subjects into two behavioral areas (internalizing and externalizing) and resulting in a total score.
  - *Conners’ Parent Rating Scale-Revised: Long Form* (CPRS-R:L).

This study was performed according to ethical principles for medical research involving human subjects stated in the Declaration of Helsinki and was approved by the local ethics committee.

We also reviewed the literature from January 2012 to February 2023 related to all published patients with *SLC6A1* pathogenic variants. We identified a total of 204 patients carrying *SLC6A1* genetic variants. The search strategy included the terms “*SLC6A1*” *and/or* “Epilepsy” on PubMed. References were also identified manually from relevant articles and by searching through the authors’ files. We limited the review to 50 English-language articles. We excluded patients with microdeletions encompassing *SLC6A1* and extended the search to other genes that could be causative of misleading data.

For all reported patients, we collected the genetic variants (including encoded protein, type of mutation and inheritance) and clinical data (including gender, age at the study, seizure types and syndromic classification, cognitive and behavioral features). Supplementary Table S1 shows a summary of the findings. We cannot exclude the presence of overlapping patients within this dataset due to incomplete data.

## Results

### Clinical cases

Table 1 reports the demographic, genetic, clinical, EEG, and neuroradiological features in our series of five patients.

**Table 1:** Clinical, genetic and neuropsychological data of our series.

Patient/gender	Epilepsy onset	Age at last FU	EEG findings	Brain MR	*SLC6A1* variant	First cognitive evaluation (test, age)	Last cognitive evaluation (test, age)	Behavioral disorders (CBCL scores)	Seizure frequency at last FU	ASMs (ongoing ASMs)
#1/M	2y	25y	Poor BA; generalized spike-and-wave complexes	Normal	c.695G > T (p.Gly232Val)	Borderline IQ 80 (Leiter-R, 7y)	Mild disability IQ 67 (Leiter-R, 12y)	Attention deficit, stereotyped behavior CBCL n.a.	Seizure-free (no ASMs)	VPA, ETS, CNZ (none)
#2/M	6y	18y	Generalized spike-and-wave complexes at 3hz (absences)	Normal	c.1070C > T (p.Ala357Val)	Borderline IQ 74 (RPM,13y)	Moderate disability IQ 44 (WAIS-IV, 18y)	Attention deficit, anxiety CBCL: INT = 75; EXT = 76; TOT = 77	Daily	ETS, CBZ, (VPA, CLB)
#3/F	3y	6y	Poor BA; bilateral sporadic spikes (mainly over T-O regions)	Normal	c.209_210insC (p.Tyr72LeufsTer135)	Normal DQ 88 (GMDS, 3y2m)	Borderline IQ 77 (WPPSI III, 5y9m)	Mild Attention deficit CBCL: INT = 43; EXT = 42; TOT = 39	Daily	CBZ, (VPA, CLB)
#4/F	6y5m	6y5m	Poor BA; slow and epileptic abnormalities on bilateral O regions	Normal (cyst of pineal gland)	c.959C > T (p.Ser320Phe)	n.a.	Borderline IQ 70 (RPM, 6y5m)	Attention deficit, disruptive behavioral disorder CBCL: INT = 67; EXT = 54; TOT = 74	Weekly	LEV, LTG, CNZ, (VPA)
#5/M	3y	6y6m	Spikes over bilateral F regions	n.a.	c.913G > A (p.Ala305Thr)	n.a.	Borderline IQ 80 (WISC IV, 6y6m)	Mild attention deficit CBCL: INT = 66; EXT = 59; TOT = 63	Seizure-free	(VPA)

M, Male; F, Female; y, years; m, months; n.a., not available; FU, follow-up; DQ, Developmental Quotient; IQ, Intellectual Quotient; ASMs, anti-seizure medications; BA, background activity; F, frontal; T, temporal; O, occipital; INT, internalizing scale; EXT, externalizing scale; TOT, total score; WAIS-IV, Wechsler Adult Intelligence Scale fourth edition; RPM, Raven’s Progressive Matrix; WPPSI-III, Wechsler Preschool and Primary Scale of Intelligence third edition; GMDS, Griffiths Mental Development Scales; WISC IV, Wechsler Intelligence Scale for Children, fourth edition; CBCL, Child Behavior Checklist; ASMs, anti-seizure medication; VPA, valproate; ETS, ethosuximide; CNZ, clonazepam; CLB, clonazepam; CBZ, carbamazepine; LTG, lamotrigine.

### Case 1

This is a 25-year-old male with normal psychomotor development before seizure onset. Epilepsy started at the age of 2 years and 3 months with atonic seizures, followed by absences with eyelid myoclonia, myoclonic-atonic, and myoclonic seizures. He was treated with valproate (VPA), ethosuximide (ETS), and clonazepam (CNZ). The last seizure was at the age of 3 years and 7 months; therefore, in adolescence, anti-seizure medications (ASMs) were discontinued without seizure relapse.

At the age of 7 years, the Leiter-R scale, exploring the nonverbal cognitive profile, revealed a borderline cognitive level (IQ = 80). At the age of 12 years, we performed a second cognitive evaluation, which detected mild cognitive impairment (IQ = 67). He attended school with a support teacher. He also had a behavioral disorder, including attention deficit and stereotyped behavior. At the age of 25 years, at the last follow-up (FU), he had mild ID requiring support in personal autonomy and self-care.

### Case 2

This is an 18-year-old male. Epilepsy started at the age of 6 years with episodes of absences with and without eyelid myoclonia. At the age of 10 years, he presented with tonic seizures, and by the age of 12, he started to present also with atonic seizures. He was treated with several ASMs, such as VPA, ETS, carbamazepine (CBZ), and clobazam (CLB), with poor efficacy. Tonic and atonic seizures have been controlled with ASMs since the age of 15 years, while daily absences persist at the last FU.

Independent gait was reached at the age of 18 months. He had speech delay: first words at 24 months; therefore, speech therapy was started.

The first neuropsychological evaluation was performed at the age of 13 years using the nonverbal cognitive test PM 38, which detected borderline intellectual functioning in terms of fluid reasoning (IQ = 74). In addition, the CBCL showed several difficulties in attention, anxiety, and social domains. He also had difficulties in managing his emotional states, which often led to behavioral dysregulation. A rehabilitation program was indicated.

We re-evaluated his cognitive performance at the age of 16 years with the PM 38. We found cognitive regression (IQ = 62), indicating mild cognitive delay. He needed school support and a differentiated school program.

Finally, we evaluated the patient at the age of 18 years with the WAIS-IV, which showed a moderate cognitive delay (IQ = 44), with greater impairment in the working memory index. He also presented with anxiety, relational disorders, and depressive features, which may have affected cognitive assessments.

### Case 3

This is a 6-year-old girl born at 35 weeks of gestation from a twin cesarean section. Independent walking was reached at the age of 16 months. Parents reported speech difficulties since the second year of life. Epilepsy onset was at the age of 3 years with atonic seizures and episodes characterized by psychomotor arrest and oral automatisms. Seizures were treated with CBZ, which worsened atonic seizures; after the replacement of CBZ with VPA and CLB, she reached complete remission of seizures.

The first neuropsychological evaluation was performed at the age of 3 years and 2 months with GMDS, which showed a developmental age of 32.5 months versus a chronological age of 37 months (DQ = 88): lower scores were seen in language and oculomotor coordination.

A second evaluation was performed at the age of 4 years and 4 months. The PM47 nonverbal cognitive scale showed an IQ of 90, with weakness in the areas of attention skills; this was not confirmed by the CPRS-R:L.

Finally, we evaluated the patient at the age of 5 years and 9 months with the WPPSI III scale, which showed borderline intellectual functioning (IQ = 77), with greater impairment in the processing speed index. We observed a deficit in attentional skills, with worsening of both selective and sustained attention.

She had been treated with psychomotor and speech therapies since the age of 3 years. Currently, the child attends primary school with a support teacher.

### Case 4

This is a 6 years and 7 months old girl born from cesarean section after a normal pregnancy. At the age of 6 years and 5 months, she presented with epileptic seizures: TCS and absences with eyelid myoclonia were reported. She was treated with levetiracetam (LEV), lamotrigine (LTG) and CNZ with poor efficacy, while VPA led to significant seizure reduction.

Motor development was normal in the first few years of life, except for delayed speech (first words at the age of 3 years) and phono-articulatory difficulties. At the age of 6 years and 5 months, she was assessed with the PM47 nonverbal cognitive scale (language barrier was clear), and she had an IQ of 70. At the same age, she also presented with behavioral disturbances characterized by oppositionality, emotional instability, heteroaggressiveness, attention deficit, and impulsivity. Poor frustration tolerance and fatigability were observed during the evaluation. Moreover, the use of CNZ had a negative impact on attention and behavior, inducing irritability, which was restored with drug withdrawal.

The child is not currently undergoing rehabilitation treatment due to poor familial psychosocial conditions.

### Case 5

This is a 6-year- and 6-month-old boy born after a normal pregnancy. His mother suffered from generalized epilepsy with absences since the age of 6 years. Neuropsychological development was normal during infancy. Epilepsy started at the age of 3 years with atonic seizures and absences with eyelid myoclonia successfully treated with VPA.

The WISC IV at the age of 6 years and 6 months revealed a borderline cognitive level (IQ = 80) with strengths in processing speed and visual spatial index and weaknesses in working memory index. In addition, we detected a mild language delay. Over time, he also developed behavioral disturbances such as attention and executive function deficits, mild anxiety, and depression.

### Genetic findings

**Figure 1:**
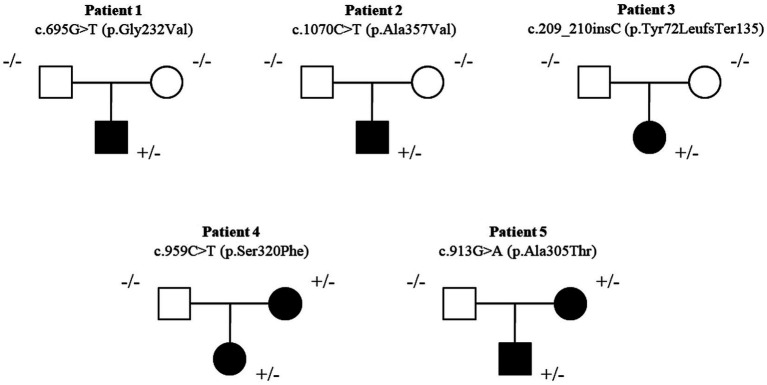
Pedigree of the five new patients. Three patients carried a *de novo* heterozygous missense variant [c.695G > T (p.Gly232Val), c.1070C > T (p.Ala357Val), c.209_210insC (p.Tyr72LeufsTer135)] and two patients carried maternal inherited genetic variant [c.959C > T (p.Ser320Phe); c.913G > A (p.Ala305Thr)] in the *SLC6A1* gene.

Three patients carried a *de novo* heterozygous missense variant [c.695G > T (p.Gly232Val), c.1070C > T (p.Ala357Val), c.209_210insC (p.Tyr72LeufsTer135)], and two patients carried a maternally inherited genetic variant [c.959C > T (p.Ser320Phe); c.913G > A (p.Ala305Thr)] in the *SLC6A1* gene (Figure 1). Patient 1, carrying c.695G > T (p.Gly232Val) genetic variant, was previously reported (Johannesen et al., 2018). Three genetic variants [c.695G > T (p.Gly232Val), c.1070C > T (p.Ala357Val) and c.913G > A (p.Ala305Thr)] were previously reported (Johannesen et al., 2018; Kim et al., 2019; Truty et al., 2019; Goodspeed et al., 2020; Kahen et al., 2022).

### Current insights from a literature review on *SLC6A1*-related disorders

We collected 204 patients with *SLC6A1* pathogenic variants. Sex was reported in 140/204 (68.6%) patients: 75 (53.6%) were females.

*SCL6A1* pathogenic variants were characterized as missense in 161/204 (79%), nonsense in 13/204 (6.3%), frameshift in 13/204 (6.3%), splice site in 10/204 (5%), inframe deletions in 5/204 (2.4%), and copy number variants (CNV) in 2/204 (1%) patients. Inheritance was available in 118/204 patients: 98/118 (83%) had a *de novo* genetic variant, and 20/118 (17%) inherited the genetic variant from parents.

Epilepsy was reported in 169 subjects. Data on seizure types were available in 116/169 patients. Different seizure types were reported: absences with or without eyelid myoclonia (94/116), atonic (70/116), myoclonic (58/116), tonic–clonic (22/116), and focal (9/116) seizures, often coexisting in the same patient. Syndromic classification was possible for 64 out of 169 patients: EMAtS was reported in 48 patients, childhood absence epilepsy (CAE) in 15 patients and LGS in 1 patient.

Neuropsychological assessments were sparsely reported in the literature, although data were often neither detailed nor standardized.

Data on neurodevelopment were available in 119/204 patients (58.3%). Developmental regression was seen in 15/119 (12.6%) cases, sometimes evolving into DD or ID. DD or ID was reported in 98/119 (92.3%) patients, subclassified as mild (27), moderate (24), or severe (8).

Eleven out of 110 (10%) subjects had normal IQ, and 1/110 (0.9%) had borderline intellectual functioning. In addition, learning disorders were mentioned in 5/11 patients with normal IQ.

Regarding psychiatric features, ADHD was reported in 20 patients, ASD or autistic features in 64 patients, and behavioral disorders in 41 patients.

## Discussion

The clinical phenotype associated with *SLC6A1* pathogenic variants includes epilepsy with various seizure types (absences, myoclonic, atonic, and tonic–clonic seizures), neurodevelopmental disorders (i.e., ASD and ADHD), and psychiatric features affecting mood and behavior (i.e., aggressivity and anxiety). To date, few data on neuropsychological features have been reported; however, data on cognition and behavioral disturbances are often missing because of a lack of standardized testing.

Developmental delay has been frequently reported by parents even before epilepsy onset, as in three out of five of our patients (Cai et al., 2019; Goodspeed et al., 2020). Once epileptic seizures appear, those patients are referred to pediatric neurologists, and soon after cognitive evaluations, a wide spectrum of cognitive and behavioral disturbances become evident (Johannesen et al., 2018; Goodspeed et al., 2020).

Data from the literature review report developmental regression in approximately 13% of patients around the age of 3 years (Kalvakuntla et al., 2023). The disability involved several domains, including language, motor, social and adaptive skills (Kalvakuntla et al., 2023). Developmental regression may be underestimated as most of the evaluations were done through unstructured interviews with parents, and standardized tests were rarely reported.

Additionally, the severity of ID was also seldom detailed in the literature, and when reported, it ranged from mild to moderate in most cases.

In general, no significant differences, in terms of cognitive impairment, were reported as related to seizure control (Johannesen et al., 2018). Accordingly, one patient in our series achieved seizure control around the age of 4 years; however, cognitive disability associated with poor autonomy in activities of daily living persisted into adulthood.

Eleven patients with normal IQ were reported in the literature, even if learning disorders were observed in five of them. Six out of 11 patients with normal IQ had epilepsy and interictal epileptiform discharges at EEGs: these findings support that etiology, rather than epilepsy, has a major role in this condition (Johannesen et al., 2018; Kim et al., 2019; Posar and Visconti, 2019; Goodspeed et al., 2020). Most of our patients had speech disorders before seizure onset, making this condition truly developmental and epileptic encephalopathy (DEE), in which epilepsy may worsen cognitive functions, which are already damaged by the genetic variant itself. The spectrum of *SLC6A1* genetic variants encompasses wide phenotypic variability, from epilepsy with or without ID to the DEE phenotype.

Our patients received several cognitive and behavioral tests. At the last neuropsychological assessment, three patients had borderline intellectual functioning, one had mild cognitive impairment, and the last patient had moderate cognitive disability. Three out of five had a worsening of cognitive functions over time. Patient 1 moved from borderline functioning to mild cognitive impairment, patient 2 from borderline to moderate cognitive delay, and patient 3 from normal to borderline functioning over 2 years without developing a clear-cut cognitive disability. What was seen in most patients was a delay in acquiring new skills rather than a clear regression. Adult patients achieved personal independence in daily activities, even if they live with their parents. They do not have jobs, and one of them still attends school with a support teacher and an individualized learning plan. They do not practice any rehabilitative or psychotherapeutic therapy. They achieved personal independence in daily activities with minimal supervision.

These findings underline the importance of longitudinal assessments in patients with epilepsy over time, especially in children of developing ages. Accordingly, periodic cognitive evaluations with standardized testing, jointly with structured behavioral assessments, might be useful to adapt over time for a patient-specific rehabilitation program to identify the best cognitive functioning and reach good daily life autonomies during adulthood.

Regarding behavioral disorders, data from the literature may be misleading. Few authors have properly analyzed these features, reported as aggressive or oppositional behaviors with psychiatric traits (Rauch et al., 2012; Lucariello et al., 2016; Snoeijen-Schouwenaars et al., 2018; Kim et al., 2019).

Consistent with literature data (Poliquin et al., 2021), we found an attention deficit disorder in all analyzed patients together with high levels of anxiety, social and disruptive behavior disorder with a pattern of oppositionality, emotional instability, and heteroaggressiveness. No pharmacological therapy was prescribed for such symptoms. Worthy of mention is the possible role of some ASMs in worsening cognitive abilities and behavioral disturbances (Strzelczyk and Schubert-Bast, 2022). Indeed, among them, drugs such as benzodiazepines may have a negative impact on attention and behavior, provoking irritability, as occurred in one of our patients.

Several studies have reported that *SLC6A1* patients with autism, from traits to ASD, are frequently related to developmental regression (Kalvakuntla et al., 2023). In particular, autism was reported in approximately one-third of patients (31.4%); in our series, one patient presented with autistic traits. We did not test patients with ASD-specific scales; therefore, the prevalence of ASD in these patients may be underestimated.

The main limitation is the small size of the sample and the lack of longitudinal data. Regarding the literature review, the unavailability of a single common database for all published cases and the lack of many details may have introduced some bias in the evaluation. In addition, considering the retrospective nature of this study, our data are heterogeneous in terms of age, timing/frequency and type of testing. We used different tests depending upon age and language barriers. It should be considered that RPM and Leiter- R, being two nonverbal cognitive tests, may overestimate patients’ IQ, unlike the Wechsler scales, which provide full IQ.

Despite the limited number of patients and the few neuropsychological data reported in the literature, the overall data suggest the need for a specific assessment for ADHD, ASD and cognitive development in patients with epilepsy related to *SLC6A1* pathogenic variants.

The high presence of behavioral disturbances and reduced cognitive performance underline the need for a targeted rehabilitation program, including strengthening attention skills.

Finally, early integrated intervention, including speech, psychological and psychomotor therapies during the early years of life, may be crucial to improve cognitive outcomes and support patients’ daily life autonomy in adulthood.

The deep phenotyping of *SLC6A1*-related disorders, including cognitive and behavioral comorbidities, is also important considering the promising pharmacological perspectives as hypothesized *in vivo* and *in vitro* studies, using chemical and pharmacological chaperones as possible treatments in deficiency of GAT-1 expression and function (Kasture et al., 2023).

## Data availability statement

The datasets presented in this study can be found in online repositories. The names of the repository/repositories and accession number(s) can be found in the article/Supplementary material.

## Ethics statement

The studies involving human participants were reviewed and approved by the Ethic Committee of Bambino Gesù Children’s Hospital. Written informed consent to participate in this study was provided by the participants’ legal guardian/next of kin. Written informed consent was obtained from the individual(s), and minor(s)’ legal guardian/next of kin, for the publication of any potentially identifiable images or data included in this article.

## Author contributions

MT, AB, AD, and CC collected and analyzed clinical data. AD and AN provided and analyzed genetic data. CQ and SC collected and analyzed neuropsychological data. MT, AB, and CQ wrote the first draft of the manuscript. SC, NS, FV, and AN supervised the study and reviewed the last version of the manuscript. All authors read and approved the submitted version.

## Funding

This work was supported by #NEXTGENERATIONEU (NGEU) and funded by the Ministry of University and Research (MUR), National Recovery and Resilience Plan (NRRP), project MNESYS (PE0000006)—a multiscale integrated approach to the study of the nervous system in health and disease (DN. 1553 11.10.2022). This work was supported by the Italian Ministry of Health with Current Research funds.

## Conflict of interest

The authors declare that the research was conducted in the absence of any commercial or financial relationships that could be construed as a potential conflict of interest.

## Publisher’s note

All claims expressed in this article are solely those of the authors and do not necessarily represent those of their affiliated organizations, or those of the publisher, the editors and the reviewers. Any product that may be evaluated in this article, or claim that may be made by its manufacturer, is not guaranteed or endorsed by the publisher.
